# Warming degrades nutritional quality of periphyton in stream ecosystems: evidence from a mesocosm experiment

**DOI:** 10.1093/ismeco/ycaf051

**Published:** 2025-03-23

**Authors:** Zhenglu Qian, Feng Zhu, Xiang Tan, Quanfa Zhang

**Affiliations:** Key Laboratory of Aquatic Botany and Watershed Ecology, Wuhan Botanical Garden, Chinese Academy of Sciences, Wuhan 430074, P. R. China; University of Chinese Academy of Sciences, Beijing 100049, P. R. China; Key Laboratory of Aquatic Botany and Watershed Ecology, Wuhan Botanical Garden, Chinese Academy of Sciences, Wuhan 430074, P. R. China; Key Laboratory of Aquatic Botany and Watershed Ecology, Wuhan Botanical Garden, Chinese Academy of Sciences, Wuhan 430074, P. R. China; Danjiangkou Wetland Ecosystem Field Scientific Observation and Research Station, the Chinese Academy of Sciences & Hubei Province, Wuhan 430074, P. R. China; Hubei Key Laboratory of Wetland Evolution & Ecological Restoration, Wuhan Botanical Garden, Chinese Academy of Sciences, Wuhan 430074, P. R. China; Key Laboratory of Aquatic Botany and Watershed Ecology, Wuhan Botanical Garden, Chinese Academy of Sciences, Wuhan 430074, P. R. China; Danjiangkou Wetland Ecosystem Field Scientific Observation and Research Station, the Chinese Academy of Sciences & Hubei Province, Wuhan 430074, P. R. China; Hubei Key Laboratory of Wetland Evolution & Ecological Restoration, Wuhan Botanical Garden, Chinese Academy of Sciences, Wuhan 430074, P. R. China

**Keywords:** river ecosystem, periphyton, food quality, warming, fatty acid, nutrient transfer, transcriptome

## Abstract

Periphyton, which is rich in polyunsaturated fatty acids (PUFA), serves as an indispensable high-quality basal resource for consumers in stream food webs. However, with global warming, how fatty acid composition of periphyton changes and consequent effects on their transfer to higher trophic level consumers remain unclear. By carrying out a manipulative mesocosm experiment with a 4°C increase, warming led to a significant decrease in the proportions of PUFA and Long-chain PUFA (LC-PUFA, >20 C) in periphyton from 13.32% to 9.90% and from 3.05% to 2.18%, respectively. The proportions of three PUFAs—α-linolenic acid (18:3ω3), arachidonic acid (ARA, 20:4ω6), and docosahexaenoic acid (22:6ω3)—also declined significantly (*P* < .05). Notably, the fatty acid profile of the consumer—*Bellamya aeruginosa* reflected the changes in basal resources, with a decrease in PUFA from 40.14% to 36.27%, and a significant decrease in LC-PUFA from 34.58% to 30.11%. Although algal community composition in biofilms did not significantly change with warming, significant transcriptomic alterations were observed, with most differentially expressed genes related to fatty acid synthesis in lipid metabolism and photosynthesis down-regulated. Our findings indicate that warming may hinder the production and transfer of high-quality carbon evaluated by LC-PUFA to consumers, consequently affect the complexity and stability of stream food webs.

## Introduction

The river ecosystem is one of the most dynamic and productive freshwater ecosystems [1], where organic matter cycling and energy flow occur through food webs. Two crucial components of river benthic food webs are basal resources and primary consumers. Periphyton on rocks (also called epilithic biofilms), which are complex microbial communities composed of algae, bacteria, fungi, and protozoa, are a vital part of river ecosystems [2, 3]. Periphyton is considered a higher-quality food source compared to terrestrial organic matter [4]. The algae in periphyton serve as significant primary producers in river ecosystems, transferring energy and carbon to higher-level aquatic consumers in the food chain and making substantial contributions to the food webs of rivers and surrounding areas [5]. *Bellamya aeruginosa*, a gastropod scraper, is ubiquitous in the aquatic ecosystems of the Yangtze River Basin, Yellow River Basin, and Yunnan-Guangxi Plateau in China [6]. As a key benthic species, it mostly feeds on periphyton, supports fish and other predators, and facilitates energy flow and nutrient cycling within the aquatic ecosystems [7].

Fatty acids, the main components of lipids, are universally present in all organisms. Polyunsaturated fatty acids (PUFA), such as arachidonic acid (ARA, 20:4ω6), eicosapentaenoic acid (EPA, 20:5ω3), and docosahexaenoic acid (DHA, 22:6ω3), influence various physiological activities of animal consumers, including regulating cell membrane fluidity, promoting the growth and reproduction of somatic cells, and serving as a form of energy storage [8, 9]. However, animal consumers are generally unable to synthesize PUFA or have limited ability to synthesize them [10] and, in most cases, cannot interconvert them or possess only limited conversion capabilities [4, 8]. Although some organisms can synthesize long-chain PUFA (LC-PUFA), the metabolic cost of this process is high [10]. In streams and rivers, primary consumers obtain PUFA by ingesting periphyton, which not only have a higher fatty acid content but also provide a more concentrated source of PUFA for fish [11].

The composition and content of PUFA have been widely used to assess food quality [4, 9] and to evaluate the trophic transfer status of ecosystems [12, 13]. Recent studies have emphasized that food quality is critical to the growth and reproduction of consumers [4] and is a key factor in determining the efficiency of energy and nutrient transfer within ecosystems [14]. Bacillariophyta, which often contain high proportions of EPA or DHA, are considered high-quality food resources [15]. In contrast, Chlorophyta, which are rich in ALA (α-linolenic acid, 18:3ω3) and LIN (linoleic acid, 18:2ω6), are regarded as moderate-quality food, while cyanobacteria, typically lacking LC-PUFA [16], are considered low-quality food for benthic invertebrates. PUFA can be retained by primary consumers and eventually transferred to higher trophic levels [17]. The effects of fatty acids on the growth, development, and other aspects of benthic animals can propagate through food chains and food webs, thereby influencing the stability and diversity of entire ecosystems [18].

Warming affects food quality, with potentially severe implications for the structure and function of freshwater ecosystems [19, 20]. Recent studies have found that rising temperatures lead to a decrease in the proportion of PUFA [21, 22]. Warming can significantly alter nutrient interactions, as well as the structure and function of communities and even food webs within aquatic ecosystems [23, 24]. It also causes a notable disruption in the nutritional linkage between plankton and animals [25], undermining the stability of aquatic ecosystems by weakening the structural integrity and biodiversity of food webs [26]. Warming may weaken the resilience of communities and lead to a negative feedback loop [27]. Climate change is likely to affect the production of fatty acids, potentially having negative impacts on food webs [22]. These potential cascading effects could impact higher trophic levels, including humans [18], and may have serious implications for the overall functioning of ecosystems [12].

Currently, the indirect effects of global warming on river ecosystems, particularly concerning the food quality of primary producers and their impacts on higher trophic levels, remain unclear. Most studies have focused on changes in the community structure or biomass of producer algae [28, 29] or shifts in consumer community dynamics [30]. While a few studies have explored changes in the nutritional quality of producers and their transfer to consumers in warming environments, these have predominantly focused on freshwater plankton [20] or marine organisms [22]. The effects of warming on the fatty acid composition and transfer within river periphyton, as well as the potential mechanisms by which periphyton regulate fatty acid metabolism, remain largely unknown.

Here, we conducted a mesocosm experiment to investigate the effects of warming (a 4°C increase in temperature) on changes in the nutritional quality of periphyton in river ecosystems and the consequent bottom-up effects. Specifically, we examined how warming influences food quality of periphyton, as indicated by PUFA, from the perspectives of composition, biochemistry, and RNA-seq transcriptomics. This study tested the following hypotheses: (i) Warming will result in a shift in the fatty acid profile of periphyton, characterized by a reduction in the proportions of PUFA, particularly long-chain PUFA (LC-PUFA). (ii) These alterations in the fatty acid composition of periphyton will impact higher trophic level consumers, such as *Bellamya aeruginosa*. Specifically, we hypothesize that the altered fatty acid profile will be transmitted to these consumers, leading to a decrease in their PUFA content. This, consequently, may impair consumers at even higher trophic levels (e.g. fish or human), also decrease the original adaptation to environment in the long term. (iii) Warming will lead to substantial transcriptomic alterations in periphyton, with a notable down-regulation of genes associated with fatty acid synthesis among differentially expressed genes (DEGs).

## Materials and methods

### Experimental setup

**Figure 1:**
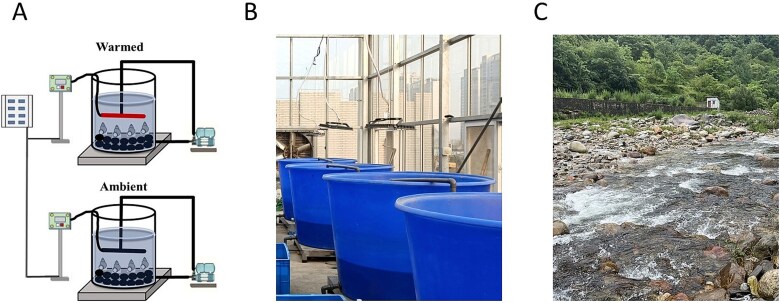
Mesocosm experiment setup (A, B), including sampling of water and cobbles with periphyton (also called epilithic biofilms) from their original habitat (C). The facility consisted of 10 fully flow-through mesocosms (volume: 1000 L; diameter: 1.2 m; height: 0.8 m). Each set of mesocosms was filled with water and supplemented with cobbles and consumers (*Bellamya aeruginosa*).

The manipulative experiment was conducted in a greenhouse at the Wuhan Botanical Garden in Wuhan, China (30°30′ N, 114°31′ E) from November to December 2022. We conducted a 35-day manipulative experiment using 10 flow-through mesocosms (volume: 1000 L, diameter: 1.2 m, height: 0.8 m). Each mesocosm was filled with 500 liters of water, and an aquarium pump was installed to circulate the water. All mesocosms were divided into two groups. We implemented a warming treatment of 4°C above ambient temperature in half of the mesocosms by maintaining a constant differential between thermocouples in paired warmed and ambient mesocosms (Fig. 1). The 4°C increase for the warmed treatment was based on IPCC Scenario AIB for temperate regions of the Northern Hemisphere [19].

In each mesocosm, the bottom was covered with cobbles (15–20 cm diameter) with periphyton, and consumers *Bellamya aeruginosa* (a snail and is very common in subtropical rivers) were added at a density of 80/m^2^ [6]. *Bellamya aeruginosa* were purchased from a local aquarium shop. After visible macrozoobenthic had been carefully removed away with forceps, the cobbles were taken to a laboratory and then were cultured to adapt to experimental conditions for 2 weeks. All the water and cobbles with periphyton were transported from the Chuka River, Macheng, Hubei Province, China (31°5′ N, 115°18′ E) (Fig. 1C).

### Sample collection and analyses

During the experiment, samples were collected each week, including water samples, periphyton samples, and consumer samples (*Bellamya aeruginosa*). At each mesocosm, three replicate samples were collected and combined, and a total of five samples from each treatment were used for replicate analysis. Surface water samples were collected separately from each mesocosm using 500 ml polyethylene bottles and stored frozen. Water temperature, pH, and dissolved oxygen (DO) were measured in situ using a portable multi-parameter water quality analyzer (YSI Professional Plus, USA). Total organic carbon (TOC), total nitrogen (TN), total carbon (TC), and other parameters were determined using a TOC/TN analyzer (Vario TOC, Germany) at the public experimental platform of Wuhan Botanical Garden. Water samples collected were filtered through pre-burned (at 450°C) 0.45 μm glass fiber filters, and the filtered water samples were used for the determination of TC, TN, and TOC [31]. Total phosphorus (TP) was measured using an inductively coupled plasma optical emission spectrometer (OPTIMA 8000DV, USA).

We randomly selected 6–8 cobbles from each mesocosm and used a hard-bristled toothbrush to scrub an area with a 10-cm diameter on each cobble. The periphyton from rocks was rinsed with distilled water into one container forming a composite sample of around 50 mL as one replicate. Additionally, the samples for identification and counting under the microscope were diluted using MQ-water (15 ml) and fixed with Lugol’s solution (1:50 dilution) [32]. For fatty acid analysis, the samples were filtered, freeze-dried, and homogenized. For transcriptomic analysis, the samples were centrifuged and stored in a freezer at −80°C.

*Bellamya aeruginosa* were collected using a type D net with a mesh size of 250 μm, and the collection process was repeated several times. The collected samples were first filtered through a 60-mesh sieve and thoroughly washed. The samples were then poured into a dissection dish for sorting, with predators and other impurities removed to ensure the purity of the collected samples. The purified samples were preserved in 50 ml centrifuge tubes and stored frozen at −20°C.

### Algal identification and numeration

Algae were identified and counted following the methods described by Hu and Wei [33], Deng [34], and Entwisle et al [35]. The community composition was determined using a microscope (Olympus BX51, Olympus Corporation, Tokyo, Japan). Before identification and counting, the samples were mixed evenly. The algal assemblages were observed under a microscope with a magnification of 400x (Zeiss Axio Imager). Counting was conducted in 100 fields of view for each sample. Based on the identification results, the proportions of the three major algal groups (Bacillariophyta, Chlorophyta, and Cyanophyta) were calculated [21].

### Lipid extraction and fatty acid profile analysis

Lipids were extracted according to a modified version of the method proposed by Vesterinen et al [36]. Samples (periphyton and *Bellamya aeruginosa*) for were freeze-dried (Freezone 4.5 L, USA), homogenized, and then re-weighed. Lipids were extracted using a 2:1 volume ratio of trichloromethane to methanol. To form fatty acid methyl esters (FAMEs), a methanolic sulfuric acid (1:100) mixture and toluene were added to the lipid extract, and the solution was incubated in a water bath at 50°C for 16 hours. FAMEs were analyzed by gas chromatography–mass spectrometry (GC–MS, Agilent 5975C).

### RNA exaction, library construction and sequencing

Total RNA was extracted from periphyton samples using a TRIzol reagent kit (Beijing, Tiangen Biochemical Technology) with three replicates for each sample. The concentration and purity of RNA were determined using a NanoDrop 2000 (Thermo Scientific, USA). Agarose gel electrophoresis (1.5%) was used to detect the integrity of the RNA (whether there was dispersion or genomic DNA contamination). Agilent 5300 Bioanalyzer (Agilent Technologies, USA) were used to determines RIN values. Only high-quality RNA sample (RIN ≥ 6.5, >1 μg) was used to construct sequencing library. Paired-end RNA-seq sequencing library was sequenced with the NovaSeq 6000 sequencing platform.

The raw paired end reads were trimmed and quality controlled by fastp (v 0.19.5). Then clean data from the samples were used to do de novo assembly with Trinity (v2.8.5). The DEGs were annotated using the COG (Clusters of Orthologous Groups), GO (Gene Ontology), and KEGG (Kyoto Encyclopedia of Genes and Genomes) databases (Fig. S1).

### Statistical analyses

The Shapiro–Wilk test was used to verify the normal distribution of the data. For non-normal data, we perform logarithmic or square root transformation. After the transformation, we checked the normality of the data again to ensure the effectiveness of the transformation method. Paired two-tailed t-tests were conducted to analyze the significance of differences in algal community composition and fatty acid data. The data were expressed as mean ± standard error, and *P* < .05 indicated statistical significance. Non-metric multidimensional scaling (NMDS) was used to analyze the impact of the temperature elevation treatment on the fatty acid composition of periphyton and *Bellamya aeruginosa*. The reliability of the NMDS results was assessed by the stress value, where a value closer to 0 indicates better model performance.

In the transcriptome data, DEGs were identified using DEGSeq2 (v1.6.3) based on RPKM (reads per kilobase of transcript per million mapped reads), with *p*-adjust <0.05 and |log_2_FC| ≥ 2 set as the thresholds to indicate significant differential expression [37]. Data analysis and graphing were performed using R 4.2.3 and Origin 2022.

**Figure 2:**
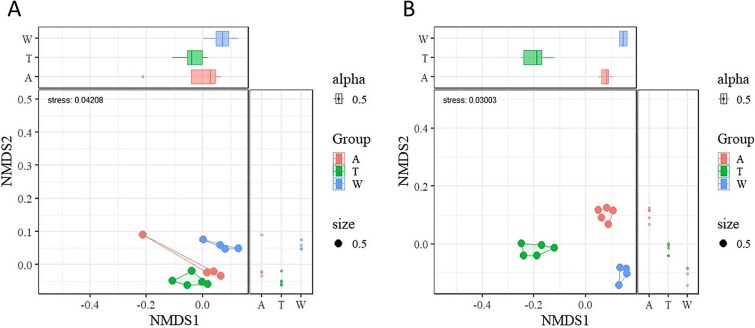
The NMDS analysis of fatty acid composition in periphyton on rocks (A) and *Bellamya aeruginosa* (B). A, ambient; W, warmed; T, initially. Stress <0.2 indicates that the model fits well.

**Table 1:** Proportion of fatty acids, including essential fatty acids (LIN, ALA, ARA, EPA, DHA) and six groups fatty acids (SAFA, MUFA, PUFA, LC-PUFA, ω3-PUFA, ω6-PUFA), in periphyton at the beginning and after 5 weeks treatment manipulative experiment (%, mean ± SE).

FAME	At the beginning	After 5 weeks treatment		T test
	Ambient/warmed	Ambient	Warmed	
C10:0	0.27 ± 0.09	0.03 ± 0.00	0.10 ± 0.01	0.00^**^
C12:0	0.33 ± 0.16	0.30 ± 0.04	0.70 ± 0.05	0.00^**^
C13:0	0.48 ± 0.16	0.08 ± 0.01	0.23 ± 0.02	0.00^**^
C14:0	7.69 ± 0.36	3.54 ± 0.25	4.42 ± 0.24	0.04^*^
C15:0	5.08 ± 0.22	5.09 ± 0.19	10.56 ± 0.18	0.00^**^
C16:0	40.03 ± 2.08	31.67 ± 0.81	26.24 ± 0.42	0.00^**^
C16:1ω7	16.38 ± 1.31	12.00 ± 0.45	16.33 ± 0.87	0.00^**^
C17:0	0.76 ± 0.11	0.57 ± 0.04	2.11 ± 0.17	0.00^**^
C17:1ω7	0.92 ± 0.10	1.38 ± 0.29	1.61 ± 0.03	0.5
C18:0	4.44 ± 0.51	3.68 ± 0.11	3.47 ± 0.09	0.19
C18:1ω9	12.83 ± 1.68	23.55 ± 0.70	18.30 ± 0.59	0.00^**^
LIN (C18:2ω6)	2.01 ± 0.38	5.04 ± 0.26	4.91 ± 0.41	0.8
C20:0	0.00 ± 0.00	0.01 ± 0.00	0.00 ± 0.00	0.05
ALA (C18:3ω3)	1.45 ± 0.50	6.52 ± 0.45	3.98 ± 0.68	0.02^*^
C20:1ω9	0.29 ± 0.11	0.79 ± 0.09	1.03 ± 0.15	0.18^*^
C22:0	0.96 ± 0.12	1.68 ± 0.12	3.04 ± 0.34	0.00^**^
ARA (C20:4ω6)	0.56 ± 0.32	0.40 ± 0.09	0.13 ± 0.02	0.04^*^
EPA (C20:5ω3)	1.41 ± 0.27	0.84 ± 0.06	0.64 ± 0.07	0.07
C24:0	0.95 ± 0.21	1.80 ± 0.12	1.80 ± 0.17	0.99
C24:1ω9	2.11 ± 1.42	0.51 ± 0.10	0.14 ± 0.02	0.01^**^
DHA (C22:6ω3)	1.06 ± 0.45	0.52 ± 0.05	0.22 ± 0.06	0.01^**^
SAFA	60.99 ± 2.43	48.46 ± 1.12	52.69 ± 0.68	0.02^*^
MUFA	32.53 ± 2.20	38.23 ± 0.55	37.42 ± 0.47	0.32
PUFA	6.48 ± 0.90	13.32 ± 0.72	9.90 ± 0.10	0.03^*^
LC-PUFA	5.43 ± 0.88	3.05 ± 0.25	2.18 ± 0.15	0.02 ^*^
ω3-PUFA	3.91 ± 0.25	7.88 ± 0.52	4.85 ± 0.55	0.01 ^**^
ω6-PUFA	2.57 ± 0.16	5.44 ± 0.24	5.05 ± 0.31	0.42

SAFA, saturated fatty acids; MUFA, monounsaturated fatty acids; PUFA, polyunsaturated fatty acids; LIN, linoleic acid (18:2ω6); ALA, α-linolenic acid (18:3ω3); ARA, arachidonic acid (20:4ω6); EPA, eicosapentaenoic acid (20:5ω3); DHA, docosahexaenoic acid (22:6ω3). ^*^P < .05, ^**^P < .01.

## Results

### Environmental characteristics of water in mesocosms

Compared to the initial stage, the physicochemical indicators of water quality changed after a five-week warming treatment. At the end of the warming treatment, the values of physicochemical indicators in the warmed groups were generally higher than those in the ambient groups (Table S1). After five weeks of warming treatment, the values of TOC, TN, TP, and DO in both the warmed and ambient groups were higher than those at the initial stage of the experiment, with the mean concentrations of TN and TP being more than twice the initial levels. Except for DO, the values of TOC, TN, TP, and pH in the warmed group were higher than those in the ambient group.

### Fatty acid profiles

The NMDS showed significant differences in the fatty acid composition of periphyton and *Bellamya aeruginosa* between the different treatment groups (Fig. 2).

**Figure 3:**
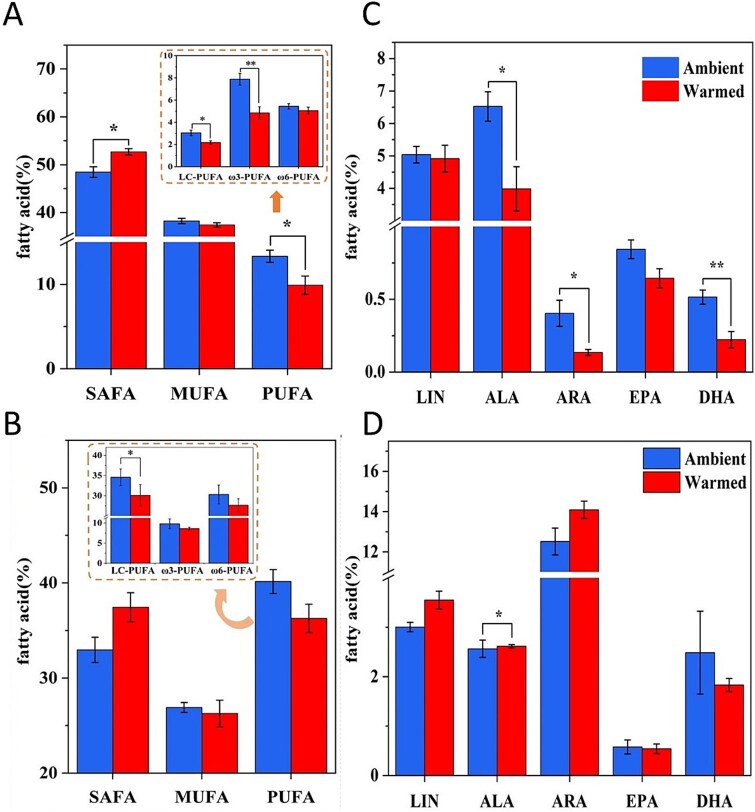
Changes in the proportion (percentages of the total fatty acids, mean ± SE, n = 5) of fatty acids in periphyton (A, C) and snails (B, D). SAFA, saturated fatty acids (fatty acids without double bonds); MUFA, monounsaturated fatty acids(fatty acids with one double bond); PUFA, polyunsaturated fatty acids (fatty acids with at least two double bonds, including ω3-PUFA and ω6-PUFA); LC-PUFA, a subclass of long-chain (≥ 20 C) PUFAs that contain more than 2 double bonds; ω3-PUFA, PUFA with the first double bond on the third carbon atom from the terminal methyl group; ω6-PUFA, PUFA with the first double bond on the sixth carbon atom from the terminal methyl group; LIN, linoleic acid (18:2ω6); ALA, α-linolenic acid (18:3ω3); ARA, arachidonic acid (20:4ω6); EPA, eicosapentaenoic acid (20:5ω3); DHA, docosahexaenoic acid (22:6ω3). (^*^P < .05, ^**^  *P* < .01).

#### Fatty acid profiles in periphyton

Analysis of the fatty acid composition of periphyton revealed the presence of 11 saturated fatty acids (SAFA), 5 monounsaturated fatty acids (MUFA), and 5 polyunsaturated fatty acids (PUFA) (Table 1). Compared to the beginning, the proportions of both MUFA and PUFA in periphyton had increased at the end of the experiment. However, compared to the ambient group, the warmed group was less pronounced. Meanwhile, the proportion of long-chain PUFA (LC-PUFA) decreased in both groups, the warmed group decreased more. After a five-week treatment, a significant increase in the overall proportion of SAFA was observed in the warmed group compared to the ambient group (*P* < .05), from 48.46 ± 1.12% to 52.69 ± 0.68%. Meanwhile, the proportions of MUFA and PUFA both decreased, from 38.23 ± 0.55% to 37.42 ± 0.47% and from 13.32 ± 0.72% to 9.90 ± 0.10%, respectively. Among them, LC-PUFA decreased significantly, from 3.05 ± 0.25% to 2.18 ± 0.15% (*P* < .05); ω3-PUFA decreased extremely significantly, from 7.88 ± 0.52% to 4.85 ± 0.55% (*P* < .01). The changes in PUFA were mainly attributed to the significant changes in alpha-linolenic acid (ALA), arachidonic acid (ARA), and docosahexaenoic acid (DHA), with proportions significantly decreasing from 6.52 ± 0.45% to 3.98 ± 0.68%, from 0.40 ± 0.09% to 0.13 ± 0.02%, and from 0.52 ± 0.05% to 0.22 ± 0.06%, respectively (Fig. 3A, C).

#### Fatty acid profiles in *Bellamya aeruginosa*

Analysis of the fatty acid composition of the consumers, *Bellamya aeruginosa*, revealed 10 SAFA, 5 MUFA, and 10 PUFA (Table 2). Notably, the proportion of SAFA was smaller, while the proportion of PUFA was larger (Fig. 3). Compared to the beginning, at the end of the experiment, the proportion of PUFA, especially LC-PUFA, decreased in the warmed group, while it increased in the control group. After five weeks of treatment, the warmed group exhibited similar changes in the proportions of SAFA, MUFA, and PUFA as observed in periphyton (Fig. 3B, D). Specifically, warming caused an increase in the proportion of SAFA from 32.96 ± 1.33% to 37.45 ± 1.52%, a decrease in MUFA from 26.90 ± 0.52% to 26.28 ± 1.39%, and a decrease in PUFA from 40.14 ± 1.26% to 36.27 ± 1.49%. The proportion of LC-PUFA decreased significantly (*P* < .05), from 34.58 ± 2.06% to 30.11 ± 2.65%. The proportions of two high-quality LC-PUFA, eicosapentaenoic acid (EPA) and DHA, both decreased.

**Table 2:** Proportion of fatty acids, including essential fatty acids (LIN, ALA, ARA, EPA, DHA) and six groups fatty acids (SAFA, MUFA, PUFA, LC-PUFA, ω3-PUFA, ω6-PUFA), in snails at the beginning and after 5 weeks treatment manipulative experiment (%, mean ± SE).

FAME	At the beginning	After 5 weeks treatment		T test
	Ambient/warmed	Ambient	Warmed	
C12:0	0.06 ± 0.01	0.05 ± 0.01	0.04 ± 0.00	0.28
C13:0	0.15 ± 0.04	0.12 ± 0.01	0.11 ± 0.01	0.20
C14:0	4.14 ± 0.34	3.41 ± 0.07	4.16 ± 0.56	0.23
C15:0	4.25 ± 0.38	3.11 ± 0.06	4.31 ± 0.79	0.23
C16:0	13.92 ± 4.15	14.74 ± 0.29	15.47 ± 0.30	0.13
C16:1ω7	9.65 ± 1.31	11.31 ± 0.44	10.47 ± 1.52	0.62
C17:0	3.19 ± 0.29	3.02 ± 0.06	2.96 ± 0.14	0.69
C17:1ω7	1.68 ± 0.32	1.72 ± 0.09	1.81 ± 0.09	0.50
C18:0	6.25 ± 0.51	6.11 ± 0.19	6.11 ± 0.22	0.98
C18:1ω9	2.43 ± 0.16	1.78 ± 0.06	2.02 ± 0.04	0.02^*^
LIN (C18:2ω6)	2.75 ± 0.45	3.00 ± 0.09	3.55 ± 0.18	0.04^*^
C20:0	0.39 ± 0.15	0.32 ± 0.20	0.13 ± 0.05	0.38
ALA (C18:3ω3)	2.79 ± 0.65	2.56 ± 0.17	2.62 ± 0.03	0.78
C20:1ω9	13.59 ± 1.53	11.25 ± 0.44	11.73 ± 0.34	0.42
C22:0	0.40 ± 0.31	0.59 ± 0.41	0.20 ± 0.11	0.40
C20:2ω6	4.79 ± 0.36	4.69 ± 0.24	3.46 ± 1.10	0.32
C20:3ω6	1.16 ± 0.22	0.81 ± 0.05	0.79 ± 0.08	0.79
ARA (C20:4ω6)	10.45 ± 2.15	12.52 ± 0.67	14.09 ± 0.43	0.10
C22:1ω9	0.86 ± 0.43	0.84 ± 0.48	0.24 ± 0.02	0.30
C22:2ω6	6.11 ± 2.29	5.89 ± 1.20	5.40 ± 0.93	0.76
EPA (C20:5ω3)	1.20 ± 0.26	0.58 ± 0.14	0.54 ± 0.10	0.84
C24:0	0.88 ± 0.27	1.49 ± 1.16	3.97 ± 0.12	0.12
C22:4ω6	3.57 ± 0.74	3.43 ± 0.33	0.38 ± 0.07	0.00^**^
C22:5ω3	3.71 ± 0.64	4.19 ± 0.63	3.62 ± 0.21	0.43
DHA (C22:6ω3)	1.65 ± 0.30	2.48 ± 0.84	1.83 ± 0.13	0.50
SAFA	33.63 ± 5.07	32.96 ± 1.33	37.45 ± 1.52	0.07
MUFA	28.20 ± 2.12	26.90 ± 0.52	26.28 ± 1.39	0.69
PUFA	39.17 ± 4.87	40.14 ± 1.26	36.27 ± 1.49	0.10
LC-PUFA	32.63 ± 1.74	34.58 ± 2.06	30.11 ± 2.65	0.04^*^
ω3-PUFA	9.35 ± 0.39	9.81 ± 1.20	8.61 ± 0.33	0.44
ω6-PUFA	28.82 ± 1.69	30.33 ± 2.33	27.66 ± 1.60	0.25

SAFA, saturated fatty acids; MUFA, monounsaturated fatty acids; PUFA, polyunsaturated fatty acids; LIN, linoleic acid (18:2ω6); ALA, α-linolenic acid (18:3ω3); ARA, arachidonic acid (20:4ω6); EPA, eicosapentaenoic acid (20:5ω3); DHA, docosahexaenoic acid (22:6ω3). ^*^  *P* < .05, ^**^  *P* < .01.

### The mechanisms of changes in fatty acid profiles

#### Algal composition in periphyton

**Figure 4:**
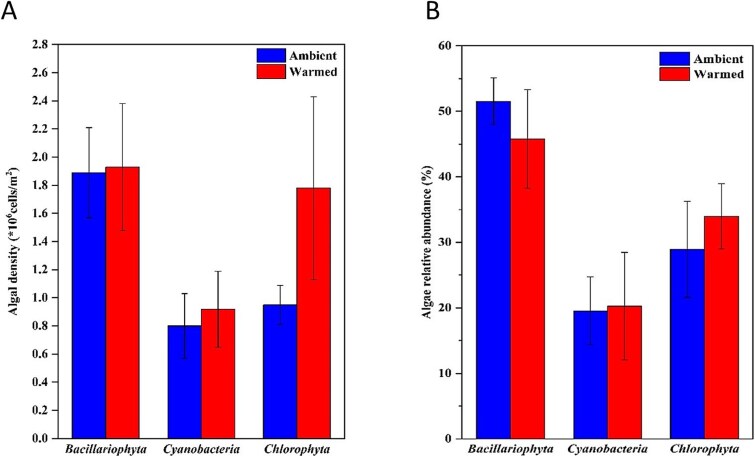
Changes in density (A) and relative abundance (B) of algal communities in periphyton at the end of the experiment (mean ± SE, n = 5). Ambient: normal water temperature; warmed: increased by 4°C.

Microscopic observations revealed that the epilithic algal community was still dominated by Bacillariophyta at the end of the experiment. Compared to the ambient group, there were no significant changes in the density and proportion of the three main types of algae (i.e., Bacillariophyta, Cyanobacteria, and Chlorophyta) in the warmed group (*P* > .05) (Fig. 4). Additionally, compared to the beginning of the experiment, there were also no significant changes observed (Table S2). However, the densities of all three algae were higher in the warmed group than in the ambient group (Fig. 4A). The proportions of Bacillariophyta, Cyanobacteria, and Chlorophyta in periphyton were 51.53%, 19.53%, and 28.93%, respectively, and warming did not significantly alter the proportions of algae (*P* > .05) (Fig. 4B).

#### Transcriptomics analysis

**Figure 5:**
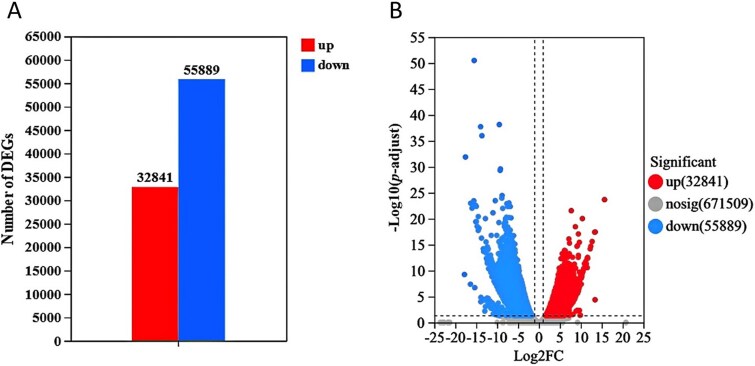
Quantity statistics bar chart (A) and volcano plot (B) of DEGs. In A, the vertical axis represents DEGs numbers, red represents the number of up-regulated DEGs (Warmed vs Ambient), and blue represents the number of down-regulated DEGs. In B, the horizontal axis indicates expression changes (log) of the genes in different treatments and the vertical axis shows the differences of gene expression. Splashes represent different genes. Gray dots are genes with no significant discrepancy, red dots are genes with significant upregulation, and blue dots are genes with significant downregulation.

**Figure 6:**
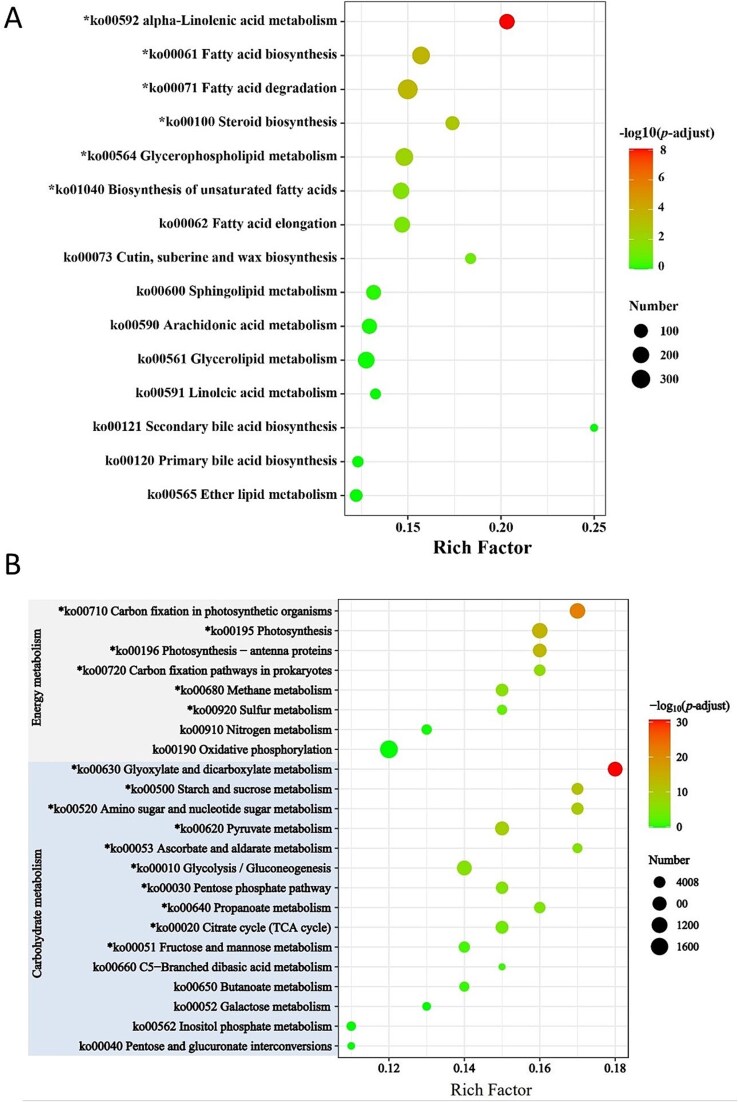
Lipid metabolism (A), carbohydrate metabolism and energy metabolism (B) enrichment analysis of DEGs. The vertical axis indicates KEGG pathway and the horizontal axis represents the rich factor. The size of bubbles indicates the number of genes in the KEGG pathway. Pathways that are significantly upregulated (red bubbles) and significantly downregulated (green bubbles). (^*^ down-regulated *P* < .05).

RNA sequencing was performed on periphyton samples. A greater number of unigenes were down-regulated in response to the temperature increase (Fig. 5). KEGG pathway enrichment analysis focused on three major functional pathways: “Lipid metabolism”, “Carbohydrate metabolism” and “Energy metabolism” (Fig. 6). Under “Lipid metabolism”, the significantly impacted pathways included “alpha-Linolenic acid metabolism”, “fatty acid degradation” and “fatty acid biosynthesis” (Fig. 6A, Table S3). Under “Carbohydrate metabolism”, “pyruvate metabolism” was observed to be down-regulated (Fig. 6B). In the “Energy metabolism” category, pathways such as “carbon fixation in photosynthetic organisms” and “photosynthesis” were also down-regulated.

**Figure 7:**
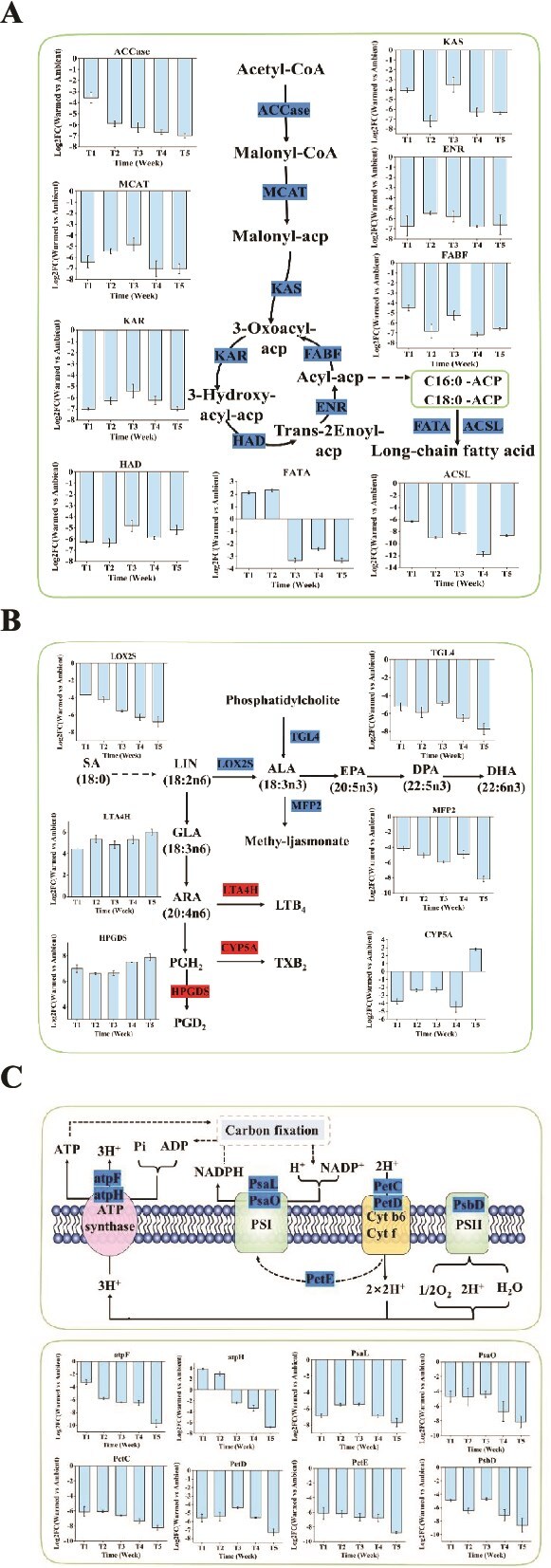
Responses of several key metabolic pathways in periphyton to warming. (A) Fatty acid biosynthesis; (B) biosynthesis of unsaturated fatty acids; (C) photosynthesis. Significantly upregulated genes (red) and downregulated genes (blue) are displayed. For abbreviations of genes names, see Table S4. The bar chart represents the changes in differentially expressed genes over a period of 5 weeks, with the vertical axis indicating the mean log_2_ (fold change) (warming vs ambient), and the error bars representing the SE (n = 3). SA (18:0), stearic acid; LIN, linoleic acid (18:2ω6); ALA, α-linolenic acid (18:3ω3); GLA (18:3ω6), γ-linolenic acid; ARA (20:4ω6), arachidonic acid; EPA (20:5ω3), eicosapentaenoic acid; DPA (22:5ω3), docosapentaenoic acid; DHA (22:6ω3), docosahexaenoic acid; PSI, photosystem I; PSII, photosystem II.

The warming treatment significantly down-regulated numerous genes related to fatty acid synthesis in the lipid metabolism of periphyton (Fig. 7A). In the fatty acid biosynthesis pathway, genes such as acetyl-CoA carboxylase (ACCase), malonyl-CoA-[acyl-carrier-protein] transacylase (MCAT), β-ketoacyl-[ACP] synthase (KAS), β-ketoacyl-[ACP] reductase (KAR), β-hydroxyacyl-[ACP] dehydratase (HAD), enoyl-[ACP] reductase (ENR), and 3-oxoacyl-[acyl-carrier-protein] synthase II (FABF) were down-regulated during the process from acetyl-CoA to acyl-[ACP]. From the first week to the end of the experiment, the warming caused a marked downregulation of ACCase, ranging from 3.55- to 7.01-fold, thereby reducing the synthesis of malonyl-CoA. Subsequently, malonyl-CoA was transferred to acyl carrier protein (ACP) by MCAT to form malonyl-[ACP]. With the involvement of corresponding enzymes, the carbon flux from malonyl-[ACP] to palmitoyl-[ACP] decreased, with downregulation ranging from 5.46- to 7.06-fold for MCAT, 4.12- to 7.20-fold for KAS, 5.39- to 7.00-fold for KAR, 4.83- to 6.35-fold for HAD, and 5.47- to 6.77-fold for ENR. In the process of converting C18:0 to long-chain fatty acids, FATA (fatty acyl-[ACP] thioesterase A) and ACSL (long-chain acyl-CoA synthase) were also significantly down-regulated. Overall, the downregulation folds (|log_2_FC|) of DEGs such as MCAT, KAS, KAR, HAD, ENR, FABF, and ACSL decreased from the first to the third week and then increased from the third to the fifth week. ACCase and FATA showed the highest fold changes in the fifth week.

The |log_2_FC| values of DEGs involved in polyunsaturated fatty acid (PUFA) biosynthesis, such as LOX2S (lipoxygenase), TGL4 (triacylglycerol lipase), MFP2 (enoyl-CoA hydratase/3-hydroxyacyl-CoA dehydrogenase), LTA4H (leukotriene-A4 hydrolase), and others, all increased over time (Fig. 7B). Specifically, LOX2S was down-regulated during the conversion of linoleic acid (LIN) to alpha-linolenic acid (ALA), ranging from 3.66- to 6.79-fold. In the processes of converting arachidonic acid (ARA) to leukotriene B4 (LTB4) and prostaglandin D2 (PGD2), genes LTA4H and HPGDS (prostaglandin-H2 D-isomerase) were up-regulated. These changes led to decreased accumulation of ARA and reduced synthesis of ALA, ultimately resulting in decreased synthesis of eicosapentaenoic acid (EPA) and docosahexaenoic acid (DHA).

In analyzing the photosynthetic metabolic pathways of periphyton, significant downregulation of DEGs was observed from the first week to the fifth week (Fig. 7C), with an increasing trend in downregulation folds. Examples include atpF (F-type H^+^-transporting ATPase subunit b), which catalyzes the formation of ATP from ADP in ATP synthase; genes PetC (cytochrome b6-f complex iron–sulfur subunit) and PetD (cytochrome b6-f complex subunit 4) in the cytochrome b6-f (Cyt b6/f) complex; the electron transfer gene PetE (plastocyanin); gene PsaO (photosystem I subunit PsaO), which catalyzes the formation of NADPH from NADP^+^ in photosystem I (PSI); and gene PsbD (photosystem II P680 reaction center D2 protein) in photosystem II (PSII). In the final week of treatment, the downregulation folds of these genes exceeded 6-fold, reaching as high as approximately 10-fold.

## Discussion

### Warming reduces the nutritional quality of periphyton and negatively impacts consumers

Periphyton, as a basal resource in rivers, is significantly impacted by environmental factors such as temperature, particularly in terms of its nutritional quality, which is often measured by the content of PUFA [3, 21]. A growing number of studies have indicated that warming can reduce the unsaturation of fatty acids [21, 38], thereby decreasing the production of essential PUFA such as EPA and DHA [39]. In this study, warming resulted in a decline in the nutritional quality of periphyton, as evidenced by reduced proportion of LC-PUFA, particularly a notable decrease in the proportion of DHA, an ω3 LC-PUFA. This observation aligns well with previous reports in phytoplankton [20, 39] and is further supported by analogous findings from earlier studies on periphyton, which collectively demonstrate the detrimental influence of temperature on PUFA content [21].

The negative correlation between temperature and PUFA accumulation [22, 39], which is prevalent in algae, is considered a mechanism for maintaining membrane fluidity [38]. As temperature fluctuates, alterations in fatty acid composition ensure the proper functioning of cellular processes and the maintenance of cellular structures [40]. Under stress conditions, such as high temperatures, algae tend to accumulate SAFA and MUFA while decreasing PUFA content, thereby preventing lipid peroxidation and maintaining cellular membrane fluidity [41]. Our findings support these observations, suggesting that the reduction of PUFA under elevated temperatures may represent a protective mechanism against thermal stress [42].

Studies have shown that the similarity in fatty acid profiles between primary consumers and periphyton varies with the PUFA content in periphyton [4]. Thus, by comparing fatty acid changes between primary consumers and producers, insights into nutrient transfer can be gained. In this study, warming resulted in a significant decrease in the proportion of LC-PUFA in *Bellamya aeruginosa*, and this change was consistent with that observed in periphyton, indicating a decrease in the nutritional quality transferred from the basal resource to the primary consumer. Furthermore, this study found that, compared to the fatty acid composition of periphyton, *Bellamya aeruginosa* had a higher content of LC-PUFA and a lower content of SAFA, aligning with previous research [43]. Typically, LC-PUFA content increases with the trophic level of organisms in stream food webs [44], whereas SAFA shows the opposite trend [45].

The reduction in LC-PUFA in algae can lead to a decline in food quality, which negatively impacts consumers [3]. Such adverse effects may include slower growth and development [10] and reduced reproductive capacity in primary consumers [46]. Through trophic transfer and relationships within the food web, the impact is anticipated to be more pronounced on consumers at higher trophic levels. Mesocosm experiments in simulated lakes have shown that EPA and DHA are essential nutrients for consumers at higher trophic levels, and their reduced availability can affect secondary production [43]. Hence, maintaining the availability of high-quality food sources rich in ω3 LC-PUFA may be crucial in mitigating the loss of freshwater biodiversity caused by global change [3].

### Mechanisms of warming effects on fatty acids in periphyton

#### Changes in algal community structure in periphyton

Specific taxonomic groups of algae have been used to characterize the composition and content of fatty acids [43, 47]. Alterations in the algal community structure within periphyton, such as a shift from Bacillariophyta to Cyanophyta and Chlorophyta, can indirectly impact the fatty acid profile of periphyton. Bacillariophyta are rich in ω3 LC-PUFA; Chlorophyta contain a higher proportion of C18 PUFA and a lower proportion of C20 PUFA; while Cyanobacteria mainly contain SAFA and MUFA [16]. The increase in the proportion of SAFA under warming treatments was related to the dominance of Cyanobacteria and Chlorophyta under these conditions [21]. Such changes in the primary producer community can potentially affect intermediate trophic levels and lead to a decline in fish nutritional quality [48, 49].

In our study, the effects of warming on the composition and density of algal communities were not significant. Conversely, temperature typically influences the composition of algal communities, with diatoms from the benthic algae thriving in colder temperatures, whereas Cyanobacteria prefer warmer environments in a previous study by Tan et al [50]. For instance, a 170-day mesocosm experiment conducted during winter revealed that Chlorophyta became more prevalent in warmed mesocosms, while Bacillariophyta were relatively abundant in unheated mesocosms [51]. The outcomes of this study might stem from the brief experimental duration (35 days).

Related studies have also indicated that under short-term warming scenarios, the direct influence of temperature on community structure is not significant [52]. The impact of warming on algal community composition varies with seasons. A recent study found differences in algal community composition between winter and summer: in summer, heated mesocosms were dominated by Cyanobacteria, while unheated mesocosms were dominated by Chrysophyta. In winter, heated mesocosms were dominated by Chlorophyta, while unheated mesocosms were dominated by Bacillariophyte and Chrysophyta [51]. Longer-duration experimental observations and the long-term adaptation of organisms to their environment may yield diverse results. However, due to climate warming, the quality of food available to organisms is expected to deteriorate. Given that there are deficiencies in current studies, it is imperative for future research to extend the experimental period and take seasonal variations into account.

#### Transcriptome analysis reveals changes in differentially expressed genes

Transcriptome analysis has significantly enhanced our understanding of how temperature regulates fatty acid biosynthesis in algae. Specifically, studies have shown that variations in temperature can affect the expression of genes involved in the fatty acid synthesis metabolic pathway in *Crypthecodinium* sp. SUN [53]. The first step in fatty acid synthesis is catalyzed by ACCase, which consumes ATP to convert acetyl-CoA into malonyl-CoA. Subsequently, malonyl-CoA serves as the carbon donor, and a series of enzymes catalyze the biosynthesis of C16:0-ACP and C18:0-ACP [54].

In this study, key genes involved in upstream fatty acid biosynthesis, including the rate-limiting enzyme ACCase, were significantly down-regulated. Other genes, such as KAS, also exhibited downregulation, suggesting a suppression in the production of C16:0-ACP and C18:0-ACP. FATA, as the chain-length-determining enzyme in fatty acid biosynthesis, catalyzes the hydrolysis of the thioester bond of acyl-ACP, releasing free fatty acids and ACP [55]. The significant downregulation of genes involved in fatty acid biosynthesis under elevated temperatures indicates a reduction in precursor substances at various stages of the fatty acid synthesis pathway, ultimately leading to decreased subsequent synthesis of PUFA.

As C16:0 and C18:0 are converted into MUFA or PUFA through a series of enzymes such as elongases and desaturases, the differential expression patterns of key genes under warming conditions provide further insights. In this study, under elevated temperature conditions, the expression of genes such as LOX2S, TGL4, and MFP2 were down-regulated with increasing fold changes over time, which suggests that the synthesis of ALA from LIN is hindered. Since ALA is a crucial precursor for other ω3 LC-PUFA, its reduced proportion in periphyton significantly limits the biosynthesis of EPA and DHA. Meanwhile, the upregulation of genes like LTA4H indicates an enhanced conversion of ARA into other compounds, such as LTB4 and PGH2, explaining the significant decrease in ARA levels in periphyton.

Photosynthesis ingeniously harnesses carbon dioxide as a substrate, converting light energy into the lifeblood of lipid biosynthesis, while concomitantly generating NADPH and ATP as the cellular energy currency, fostering lipid accumulation [56]. The increase in NADPH content through the regulation of certain genes contributes to the increased production of LC-PUFA, especially DHA [57]. Research indicated that as temperatures rise, the functional potentials of PSI and PSII in planktonic bacteria are significantly inhibited, leading to an overall decline in the activities of NADPH and ATP synthases [58]. This change restricts the metabolic potentials of electron transport chains (e.g., ferredoxin-NADP^+^ reductase) and energy generation (e.g., F-type ATPases), thereby weakening the efficiency of CO_2_ fixation and the synthesis of organic carbon [59]. In our study, multiple key genes involved in photosynthesis were significantly down-regulated, revealing the negative effects of warming on photosynthetic metabolism. This reduction led to decreased ATP and NADPH production, impacted carbon fixation in the Calvin cycle, and curtailed the availability of fatty acid precursors such as pyruvate and acetyl-CoA, ultimately limiting fatty acid synthesis.

## Conclusions

Since PUFA is indispensable to consumers but consumers almost cannot biosynthesize it, we use it two ways: as both a biomarker of high-quality carbon and a tracer to trace PUFA transfer to consumers. We found that warming significantly decreased the proportion of PUFA, one ω3-PUFA (ALA) and DHA (LC-PUFA), in periphyton while concurrently reducing the proportion of LC-PUFA in *Bellamya aeruginosa*, elucidating the transmission of adverse effects along food chains and food webs. Our study has shown that even if there are no significant changes at the macroscopic community level, microscopic metabolic pathways have already been significantly impaired. We offer a novel perspective on the implications of future warming on basal resources and good quality carbon transfer within river ecosystems, underscoring the urgent need for effective measures to address global warming. However, this indoor experiment cannot fully capture the complexities of wild river conditions. Future research should also explore how global warming affects the survival strategies of consumers in aquatic ecosystems.

## Supplementary Material

Supplementary_ZQ20250318_ycaf051

## Data Availability

Raw sequence data is deposited in the NCBI archive under accession number PRJNA1179879. All other data are available on figshare: https://doi.org/10.6084/m9.figshare.27304260.
