# Supplementary material for: Warming degrades nutritional quality of periphyton in stream ecosystems: evidence from a mesocosm experiment
Source: ISME Commun. 2025 Mar 23;5(1):ycaf051. doi: 10.1093/ismeco/ycaf051 (PMC11977459; doi:10.1093/ismeco/ycaf051)
Supplement: Supplementary_ZQ20250318_ycaf051 [file supplementary_zq20250318_ycaf051.docx]

**Table S1** Environmental characteristics of the waters in the mesocosms (mean ± SE).

|  | At the beginning | After 5 weeks treatment | | | |
| --- | --- | --- | --- | --- | --- |
|  |  | Ambient | | Warmed | |
| Temperature (°C) | 20.38±0.18 | 9.80±0.49 | 13.80±0.49 | |  |
| TOC (mg·L^-1^) | 6.35±1.30 | 11.81±1.49 | 15.63±4.46 | |  |
| TN (mg·L^-1^) | 1.54±0.42 | 3.67±0.28 | 6.02±1.83 | |  |
| TP (mg·L^-1^) | 0.07±0.01 | 0.24±0.05 | 0.33±0.08 | |  |
| DO (mg·L^-1^) | 5.70±0.18 | 5.80±0.03 | 5.74±0.12 | |  |
| pH | 8.01±0.08 | 7.80±0.10 | 8.07±0.06 | |  |

**Table S2** Taxonomic compositions of algal communities in periphyton on rocks in the mesocosms.

| Taxa in Periphyton | At the beginning | After 5 weeks treatment | |
| --- | --- | --- | --- |
|  |  | Ambient | Warmed |
| ***Bacillariophyta*** |  |  |  |
| *Achnanthes* | 0.13% | - | - |
| *Asterionella formosa* Hassall | 0.25% | - | - |
| *Aulacoseira granulata f. spiralis* (Hustedt) Czarnecki & D.C.Reinke | - | 1.98% | - |
| *Cocconeis* | - | - | 1.12% |
| *Cymatopleura* | - | 0.22% | - |
| *Cymbella* | 5.76% | 10.76% | 5.70% |
| *Diatoma* | 0.38% | 0.44% | - |
| *Diploneis puella* | 0.25% | - | - |
| *Fragilaria* | 11.08% | 12.51% | 5.35% |
| *Gomphonema* | 14.44% | 17.23% | 19.43% |
| *Gyrosigma* | 0.25% | 0.44% | 0.35% |
| *Melosira granulata* var. *angustissima* O. Müller | 6.02% | 2.41% | 1.38% |
| *Melosira varians* Agardh | 6.46% | 2.63% | 4.15% |
| *Navicula* | - | 0.66% | 0.35% |
| *Nitzschia* | 1.01% | - | 0.69% |
| *Rhoicosphenia abbreviata* (C.Agardh) Lange-Bertalot | 0.57% | 0.44% | 0.35% |
| *Surirella* | 0.38% | - | 0.35% |
| *Synedra* | 7.35% | 2.20% | 2.50% |
|  |  |  |  |
| ***Chlorophyta*** |  |  |  |
| *Ankistrodesmus acicularis* (Braun) Korschikoff | 2.15% | 3.51% | 15.89% |
| *Ankistrodesmus angustus* Bernard | 0.63% | - | 7.43% |
| *Closterium* | 0.32% | 0.44% | 0.17% |
| *Cosmarium* | - | - | 0.35% |
| *Mougeotia* | 0.25% | - | - |
| *Pediastrum* | 2.03% | - | - |
| *Pediastrum duplex* Meyen | 2.03% | 3.51% | 2.76% |
| *Scenedesmus bijuga* (Turpin) Lagerheim | 0.89% | 0.66% | 0.86% |
| *Scenedesmus dimorphus* (Turpin) Kützing | 0.63% | 7.68% | 7.69% |
| *Scenedesmus quadricauda* (Turpin) Brébisson | 0.25% | 6.59% | 2.59% |
| *Sphaerocystis schroeteri* Chodat | - | 0.44% | 0.35% |
| *Spiroggra* | 6.33% | - | - |
| *Tetraedron* | 0.38% | - | 0.35% |
| *Ulothrix* | 5.70% | 3.29% | - |
|  |  |  |  |
| ***Cyanobacteria*** |  |  |  |
| *Lyngbya* | 16.47% | 9.88% | - |
| *Planktolyngbya* | 7.60% | 6.59% | 16.41% |
| *Oscillatoria* | - | 5.49% | 3.45% |

**Table S3** Lipid metabolism in KEGG analysis of differentially expressed genes (DEGs) in periphyton on rocks.

| ko | Description | up numbers | down numbers | *p*-value | *p*-adjust |
| --- | --- | --- | --- | --- | --- |
| ko00592 | alpha-Linolenic acid metabolism | 48 | 105 | 2.65E-10 | 8.21E-09 |
| ko00061 | Fatty acid biosynthesis | 66 | 196 | 1.93E-05 | 2.62E-04 |
| ko00071 | Fatty acid degradation | 171 | 209 | 2.52E-05 | 3.21E-04 |
| ko00100 | Steroid biosynthesis | 43 | 61 | 1.67E-04 | 1.69E-03 |
| ko00564 | Glycerophospholipid metabolism | 88 | 176 | 7.78E-04 | 6.89E-03 |
| ko01040 | Biosynthesis of unsaturated fatty acids | 90 | 109 | 5.11E-03 | 3.76E-02 |
| ko00062 | Fatty acid elongation | 41 | 130 | 7.58E-03 | 5.22E-02 |
| ko00073 | Cutin, suberine and wax biosynthesis | 8 | 19 | 2.07E-02 | 1.23E-01 |
| ko00600 | Sphingolipid metabolism | 57 | 73 | 2.09E-01 | 7.67E-01 |
| ko00590 | Arachidonic acid metabolism | 103 | 39 | 2.63E-01 | 9.19E-01 |
| ko00561 | Glycerolipid metabolism | 86 | 120 | 2.81E-01 | 9.54E-01 |
| ko00591 | Linoleic acid metabolism | 11 | 13 | 3.74E-01 | 1 |
| ko00121 | Secondary bile acid biosynthesis | 0 | 1 | 4.08E-01 | 1 |
| ko00120 | Primary bile acid biosynthesis | 31 | 2 | 5.18E-01 | 1 |
| ko00565 | Ether lipid metabolism | 31 | 30 | 5.32E-01 | 1 |

**Table S4** Differentially expressed genes (DEGs) involved in fatty acid metabolism and photosynthesis in periphyton on rocks. A, ambient; W, warmed.

| Pathway | Gene name | Description | W vs A |
| --- | --- | --- | --- |
| Fatty acid biosynthesis | ACCase | Acetyl-CoA carboxylase | down |
|  | MCAT | Malonyl-CoA ACP transacylase | down |
|  | KAS | β-ketoacyl-ACP synthase | down |
|  | KAR | β-ketoacyl-ACP reductase | down |
|  | HAD | β-hydroxyacyl-ACP dehydrase | down |
|  | ENR | Enoyl-ACP reductase | down |
|  | FABF | 3-oxoacyl-[acyl-carrier-protein] synthase II | down |
|  | FATA | Fatty acyl-ACP thioesterase A | down |
|  | ACSL | Long chain acyl-CoA synthase | down |
| Biosynthesis of unsaturated fatty acids | LOX2S | Lipoxygenase | down |
|  | TGL4 | Triacylglycerol lipase | down |
|  | MFP2 | Enoyl-CoA hydratase/3-hydroxyacyl-CoA dehydrogenase | down |
|  | LTA4H | Leukotriene-A4 hydrolase | up |
|  | CYP5A | Thromboxane-A synthase | up |
|  | HPGDS | Prostaglandin-H2 D-isomerase | up |
| Photosynthesis | atpF | F-type H^+^-transporting ATPase subunit b | down |
|  | atpH | F-type H^+^-transporting ATPase subunit delta | down |
|  | PsaB | Photosystem I P700 chlorophyll a apoprotein A2 | down |
|  | PsaL | Photosystem I subunit XI | down |
|  | PsaO | Photosystem I subunit PsaO | down |
|  | PsbB | Photosystem II CP47 chlorophyll apoprotein | down |
|  | PsbD | Photosystem II P680 reaction center D2 protein | down |
|  | PetC | Cytochrome b6-f complex iron-sulfur subunit | down |
|  | PetD | Cytochrome b6-f complex subunit 4 | down |
|  | PetE | Plastocyanin | down |

**(a)**

**
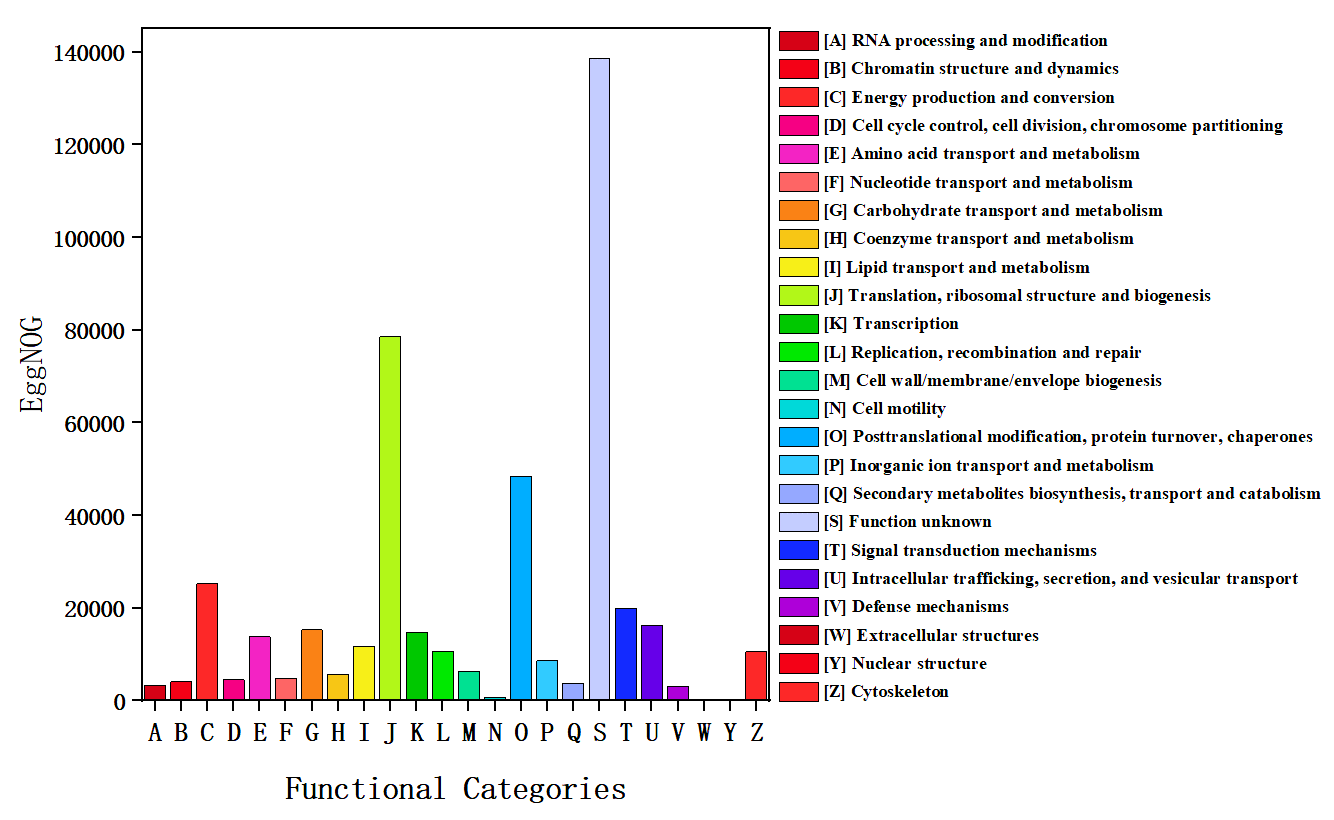
**

**(b)**


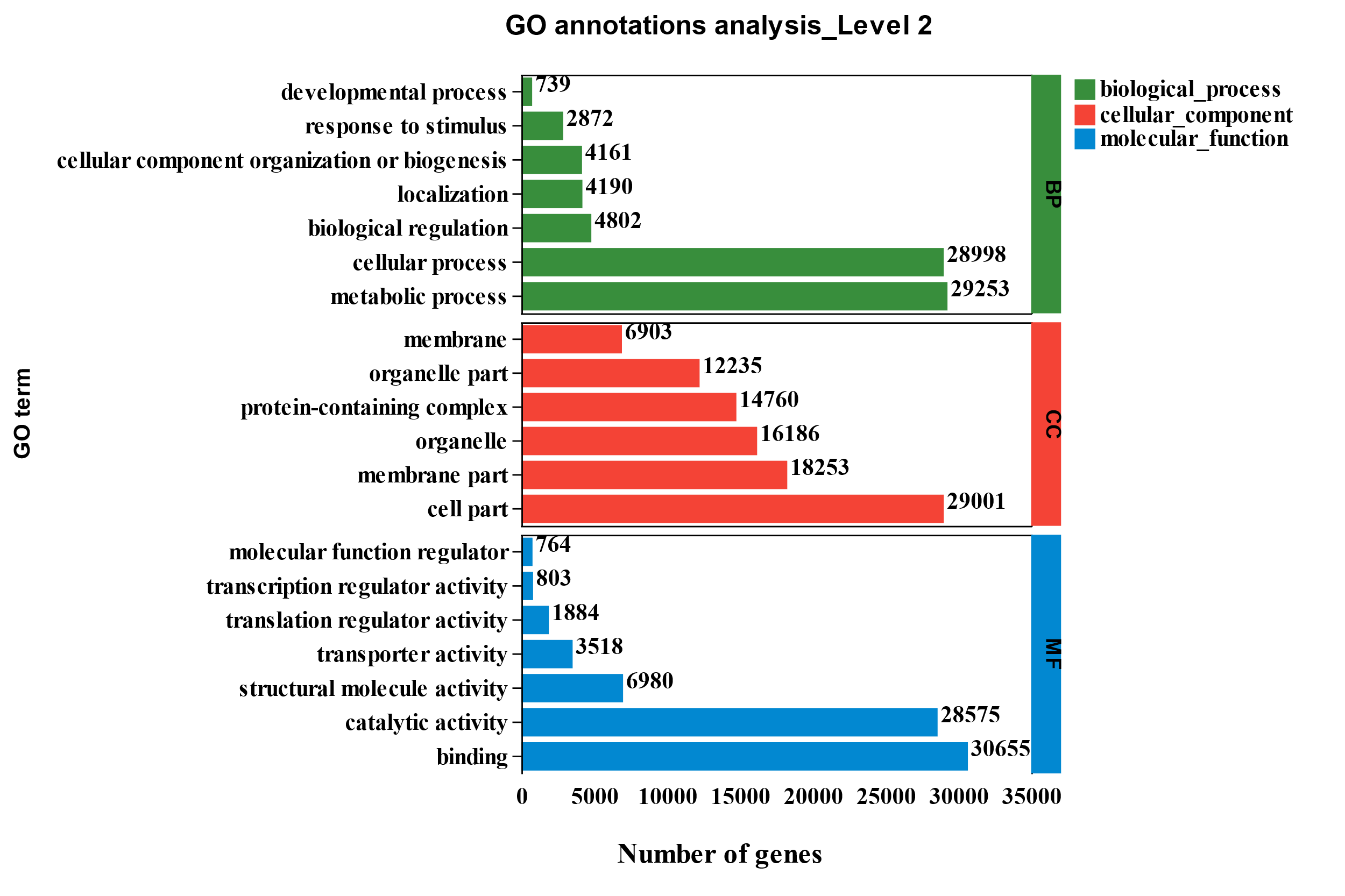


**(c)**


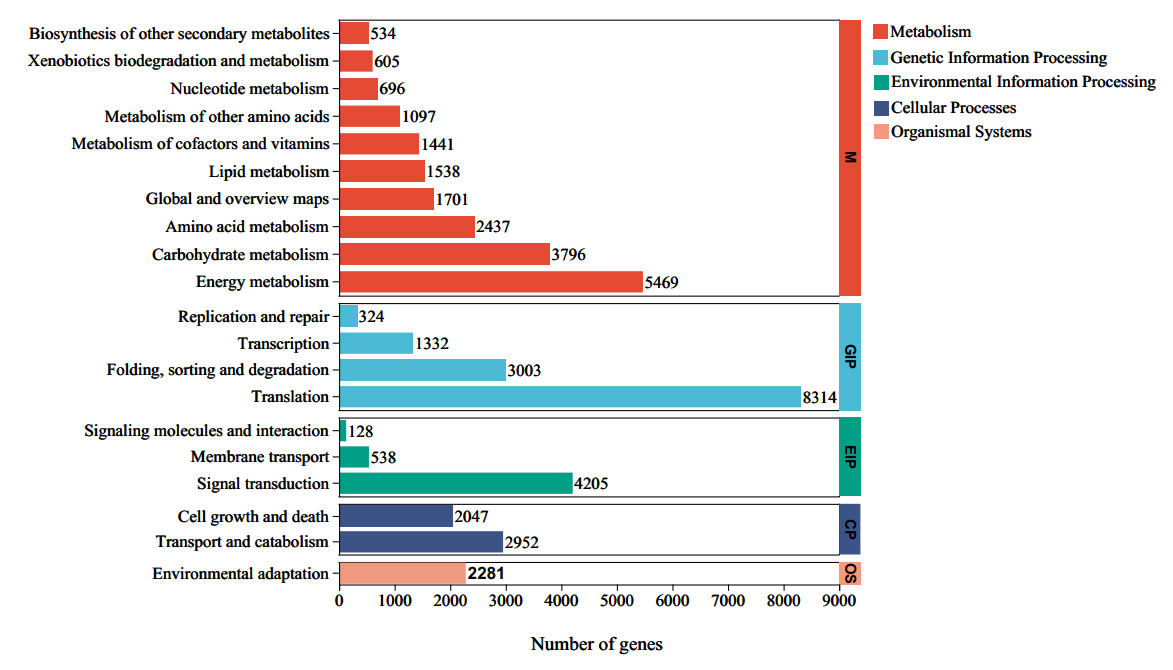
**Fig. S1** Functional annotation of differentially expressed genes (DEGs) in GO (a), COG (b), and KEGG (c) databases in periphyton on rocks.
